# Editorial: NK Cells in Human Diseases: Friends or Foes?

**DOI:** 10.3389/fimmu.2017.01737

**Published:** 2017-12-07

**Authors:** Vincent Vieillard, Bree Foley, Sandra L. López-Vergès

**Affiliations:** ^1^Center of Immunology and Infectious Diseases, Pitié-Salpêtrière Hospital, Paris, France; ^2^Telethon Kids Institution, University of Western Australia, Subiaco, WA, Australia; ^3^Gorgas Memorial Institute of Health Studies, Panama City, Panama

**Keywords:** natural killer cells, anti-microbial response, antiviral response, antitumor response, transplantation, uterine natural killer cells

**Editorial on the Research Topic**

**NK Cells in Human Diseases: Friends or Foes?**

Natural killer (NK) cells are lymphocytes from the innate immune system that play a protective role against tumors and viral infections. Activated human NK cells respond by killing infected or tumor cells and can shape the adaptive response by producing cytokines and chemokines and by interacting with dendritic cells and other immune cells, and also have memory-like features (1). For these reasons, NK cells are principally considered as protective cells. However, recent discoveries have found that NK cells can have a negative role due to overproduction of pro-inflammatory cytokines, destruction of host cells in autoimmune diseases, in graft-versus-host diseases, or in regulating other immune cells that provide protective immunity, e.g., in some cancers where NK cells counteract tumor-specific infiltrating T cells. The aim of this research topic is to analyze the different situations in which NK cells could have a beneficial or a deleterious role and what are the signals and mechanisms that could explain these differences in behavior. In this era of immunetherapy, understanding of NK-cell biology and its relation to human disease is crucial for rational design of treatments that are efficient but also are safe.

## NK Cells and Anti-Microbial Response

Natural killer-cell responses have been described historically in chronic viral infections, particularly with members of the herpes viral family. Human cytomegalovirus (CMV) infection has been shown to induce the expansion of a specific subset of peripheral blood NK cells that are adaptive-like (2). Other viral infections can also induce these adaptive-like NK cells; however, these cells seem strongly associated with CMV seropositivity (3, 4). Malone et al. investigated if these expansions could be affected by concurrent chronic hepatitis virus infections. More studies are needed to determine if these adaptive-like NK cells in CMV seropositive individuals are more responsive during all infections or only in a subset of them, or during tumor responses, and if this induces a difference in the severity of the disease. Are they as functional in patients with HIV infection? NK-cell function as well as that of other immune cells can be dysregulated during HIV infection and its inflammatory status. As the incidence of some cancers is increased in HIV-infected patients, with NK cells shaping the response to both viral infections and cancer, Leal et al. tried to differentiate anti-HIV and antitumor NK-cell features with the goal to determine if NK cells could concurrently be manipulated in the future to halt HIV disease and HIV malignancies. It is clear that NK cells play a crucial role in controlling viral load and eliminating virus-infected cells; however, they do not always respond with the same intensity to infections. The phenotype of each subset of NK cells, the different alleles of NK-cell receptors, as well as splice variants of these receptors, all play a role in determining NK-cell responses. Shemer-Avni et al. explored the predominance and the difference in activity of splice variants of the NKp46 receptor during respiratory viral infections, opening the path to their analysis during other infections in which NKp46 has been shown to play a role. Is this mechanism of regulation of receptor activity specific for NKp46 or is it general for all NK receptors?

Natural killer cells are mainly studied for their antiviral activities; however, it has been shown that they could play a role against intracellular bacteria and parasites and even against fungal infections. The role of NK cells in some parasitic infections such as malaria can vary during the course of infection, is heterogeneic, and can also be influenced by host genetics as reviewed by Wolf et al. NK cells kill or inhibit the growth of different fungi. Ogbomo and Mody discussed how NK cells recognize fungi-infected cells and the mechanisms for the induction of granule-dependent cytotoxicity.

## NK Cells and Antitumoral Response

Due to the selective pressure of NK cells in tumor development, tumors have developed many escape mechanisms, similarly to viruses, to avoid NK-cell recognition and activation (5). One of these is the regulation of the expression of the ligands of activating receptors. A better understanding of the underlying mechanisms may allow the discovery of a potential therapeutic approach. Moncayo et al. showed that MICA expression seems regulated by cell adhesion and contact and this could play a role in cancer immune evasion. Another escape mechanism could be the alteration of NK-cell behavior by the tumor microenvironment; Stabile et al. reviewed how many components of this microenvironment could influence NK cell developmental programming or the response of distinct NK-cell subsets. Different JAK/STAT pathways could be activated through these components, thus Gotthardt and Sexl discussed how this could shape NK-cell behavior. It is still necessary to determine if and how some STAT family members contribute to the switch from tumor suppression to tumor progression. Also, future studies are needed to demonstrate if by modifying or eliminating some microenvironment components or regulating some JAK/STAT pathways, it is possible to harness NK-cell subsets so they can control once again solid and hematological malignancies.

Through their cytokine and chemokine production as well as their cross talk with other immune cells, NK cells can regulate tissue inflammation and regeneration. Tosello-Trampont et al. showed that it is through this immunoregulatory role that NK cells play an important role in metabolic liver diseases.

## NK Cells and Transplantation

The role of NK cells in graft acceptance or rejection of solid tissues has been largely studied to help in determining the best host–donor match. The treatment of hematological malignancies through allogeneic hematopoietic cell transplantation is still associated with significant morbidity and mortality related to cancer relapse as well as to transplant-related complications including graft-versus-host disease (GvHD). Even if the protective role of NK cells against cancer relapse is well sustained, their role in GvHD is still controversial. Simonetta et al. reviewed and discussed this with the goal of obtaining a model that could show which are the conditions that could induce naturally suppressing NK cells to sustain GvHD. More studies are needed to determine if the adaptive-like NKG2C^+^ NK cells that play a role in the control of viral infections in HSC and solid transplant recipients contribute to control of GvHD.

## Uterine NK Cells

Natural killer cells do not only play a crucial role during disease. Uterine NK cells are required to have a healthy pregnancy, as they regulate trophoblast invasion and uterine spiral arterial remodeling in humans. Gaynor and Colucci reviewed the current knowledge on their development and function. The emergence of Zika virus, which can be transmitted from an infected-mother to the fetus resulting in strong neurological consequences for the fetus, shows the importance of studying uterine NK cells as they could play a role in Zika virus control at the level of the placental barrier.

In conclusion, we want to express our gratitude to all the authors who have contributed to this research topic and to the reviewers for their valuable work showing that many components can shape NK cell behavior and thus have consequences on human disease.

## Author Contributions

SL-V wrote the editorial; VV and BF edited the editorial.

## Conflict of Interest Statement

The authors declare that the research was conducted in the absence of any commercial or financial relationships that could be construed as a potential conflict of interest.
